# The effect of increased feeding occasions per day on the incidence of tail biting–a case report

**DOI:** 10.1186/s40813-026-00537-x

**Published:** 2026-09-29

**Authors:** Hanna Andersson, Magdalena Jacobson

**Affiliations:** 1 Möre Veterinary Service, Söderåkra, Sweden; 2 Department of Clinical Sciences, Faculty of Veterinary Medicine and Animal Sciences, SLU, Uppsala, Sweden

**Keywords:** Swine, Tail biting, Feeding regimens, Slaughter remarks, Antibiotic treatments

## Abstract

**Background:**

Tail biting is a recurring and severe problem in the pig production, being provoked by a multitude of underlying factors predisposing for an abnormal behaviour. Risk factors include feed-related factors such as restricted feeding, a high-energy, low-fibre ration, unpredicted feeding times, and competition for food. In 2010, an increase in the number of feeding bouts from three to four times a day was implemented in a Swedish finisher farm. Following this change, a decrease in the number of pigs with tail lesions being recorded at slaughter, and a drop in the number of pigs treated with antibiotics for tail biting, was noted. The aim of this study was to investigate retrospectively the effect of changing the number of feeding times per day in 15 868 pigs being bred during 15 months before (Period 1) to 15 months after (Period 2) this change was implemented, including the remarks for tail biting at slaughter, the number of pigs treated for tail biting, the daily weight gain (DWG) and the feed conversion ratio (FCR) during these periods.

**Results:**

In total 6390 pigs in Period 1, and 9478 pigs in Period 2, were included. The average number of remarks for tail lesions at slaughter was reduced from 147 pigs to 113 pigs, a reduction by 54% (*p* < 0.001). The number of pigs subject to antibiotic treatment for “tail biting” was reduced from 0.9% to 0.6%, a reduction by 35% (*p* < 0.01). The average DWG during Period 1 was 790 g/day and during Period 2, the DWG was 830 g/day (*p* < 0.0007). The FCR as measured by MJ/kg growth was 38.5 MJ/kg during Period 1, and 37.2 MJ/kg growth during Period 2 (*p* < 0.024).

**Conclusion:**

An increase in the number of feeding bouts from three to four times daily seems to reduce the frequency of tail biting, as monitored by the number of remarks at slaughter and the number of treatments for tail biting. Further, the increase resulted in a higher average daily weight gain and in a decrease in the feed conversion ratio.

## Background

Tail biting is a recurring and severe problem in the pig production, with reported prevalences ranging from 1% in tail-docked pigs at slaughter [1] to 99.3% of the undocked, conventionally reared, finisher pigs [2]. Many lesions have also healed at slaughter and may therefore not be reported [3]. Tail biting implies an animal welfare problem, cause pain to the affected animal, may cause severe secondary infections, and may affect the productivity [2, 4, 5].

Acute cases are treated with wound spray, antibiotics, non-steroidal antiphlogistic drugs, or repellents such as tar, environmental improvements, removing of the biter, and removing of the victim [6–10], measures that however may reduce the incidence but not eliminate the biting entirely [11]. Thus, the problem is commonly mitigated by tail docking [2, 3, 6, 12]. In Sweden, this praxis was banned in 1988 (Djurskyddslagen; SFS 1988:534), whereas EU banned docking in 1994 (Council Directive 91/630/EEG). However, dispensations are common and reportedly, Sweden, Finland and Lithuania are presently the only EU-member states not practising tail docking at all [1, 10, 13].

Tail biting is provoked by a multitude of underlying factors predisposing for an abnormal behaviour. Influencing factors listed in the literature include gender [6, 14, 15], breed [5, 16], genetics [17], regrouping and rearing system [2], stocking density [3, 12, 18, 19], environmental factors such as slatted floors [18, 20], air quality and ventilation [6, 21], temperature [20, 22], and noise [9]; and other diseases [18]. Further, the change of caretaker is regarded as problematic by the farmers [9, 19].

Provision of straw is the most commonly recommended measure to mitigate tail biting [6, 9] - [12, 18]). It provides a rooting material, thus fulfilling the exploratory behaviour and the appetitive foraging behaviour of the pig [23].

In growing-fattening pigs, the incidence commonly increases with weight or age [3, 4, 10, 24]. In a survey performed by Bracke [8], restricted feeding in breeding gilts was described as a predisposing factor. Finisher pigs may be fed *ad libitum* during the first half of the fattening period, but be subjected to restricted feeding during the second half [23], a practise that according to Swedish pig producers (pers. comm.) is related to tail biting. On the contrary, farmers in the UK did not consider restricted feed, feeding according to appetite or *ad lib* to affect the probability of being bitten [6]. Further, the structure of the feed (meal, liquid feeding, pelleted feed, ground grain) and the protein level may affect the frequency [6, 18, 19, 22, 25]. A high-energy, low-fibre ration will improve the digestibility, but the appetite of the pig may be unsatisfied [19]. A higher back-fat value reduced the risk of tail biting, whereas selection for lean meat might predispose [8, 18]. Unpredicted feeding times [21] and competition for food by restricted access to feeder space may lead to tail biting [4, 6, 18, 20] and may also increase with size variation, since some pigs will not be able to shoulder their way to the feeder [25]. Thus, the cannibalistic offender is frequently the runt of the group [19]. Few studies have addressed the time interval between the meals, although Hessel et al. [26] compared feeding three times a day to feeding nine times a day.

The aim of this study was to investigate retrospectively the effect of changing the number of feeding occasions per day in a Swedish finisher farm and describe the prevalence of tail biting during a period of 15 months before, to 15 months after this change was implemented.

## Material and methods

The investigations were performed in a herd raising fattening pigs for slaughter. In 2010, an increase in the number of feedings from three to four times a day was implemented in this herd. Following this change, a decrease in the number of pigs with tail lesions being recorded at slaughter, and a drop in the number of pigs treated with antibiotics for tail biting, was noted. Therefore, a study was undertaken scrutinizing the herd records during 15 months before (Period 1), to 15 months after (Period 2) the change in feeding regime. During these periods, no other changes in management or nutrition were made and the same animal caretaker were managing the pigs. The data included the number of pigs recorded with “tail lesion” at slaughter, the number of treatments for “tail biting” recorded, the average growth performance, and the average feed conversion ratio.

### Herd and management

The herd was a specialised finisher herd producing approximately 9000 pigs for slaughter per year. At any one time, the herd kept 3240 pigs allocated in nine separate stables, joined by a corridor that was separated from the stables by walls and closed doors. Each stable included 34 pens with 10–11 pigs per pen, and two additional “sick pens”. Each pen consisted of 6.2 m^2^ solid concrete floor and 2.3 m^2^ raised, slatted floor in a dunging area partly separated by a low wall. The space available at the feeding through was 31 cm/pig. Each stable was run according to all-in/all-out principles with high-pressure washing in-between each batch, receiving batches of 360 pigs at 25–30 kg b.w. and 10 to 11 weeks of age, being raised to slaughter at approximately 120 kg b.w. The pigs were weighed at pen level at arrival and individually at slaughter. Feed intake was measured daily at pen level. The pigs were bought through an agency, certified to be healthy and with intact tails, and each batch included pigs from up to seven, various, piglet-producing herds. The pigs were crossbreds between Yorkshire × Landrace dams and either Hampshire or Duroc sires. Tail-docking was, in accordance with legislation, not performed in any of these herds. The finisher herd was managed by one foreman, and one caretaker that was assigned to more simple, everyday tasks. The stables were under negative-pressure ventilation set at an initial temperature of 22 °C, being lowered to 15 °C later during rearing, and heating was provided in four underfloor heat loops and by hot water in four front pipes. Manure was removed two times daily by scrapers under the slatted floor. In January 2011 measurements of some climate parameters were performed, including ammonia (varying from 14 to 36 ppm in a stable with 22 week-old pigs, from 16 to 31 ppm in a stable with 18-week old pigs, and from 10 to 26 ppm in a stable with 14-week old pigs, as measured over the dunging area in 36 pens and altogether 3 stables), carbon dioxide (varying from 1000 to 2800 ppm), noise (varying from 62 to 82 dBA) and indoor temperature (varying from 14.3 to 18.4 °C, the outdoor temperature being −2.5°C), as measured in three stables. When the pigs arrived, the pens were bedded with wood shavings, later during rearing supplemented with straw that was provided 1–2 times daily, approx. 1 kg per pen and day. The pigs had free access to water, provided in nipple drinkers. In case of tail biting, bitten pigs were treated (see below) and the pen was provided with “toys” such as car tyres, salt licks, and short hose pipes.

### Feed

The automated feeding system started at 6 a.m. In “Period 1”, the feed was provided three times per day at seven-hour intervals, and in “Period 2”, feed was provided four times per day at six-hour intervals. The pigs were fed a liquid feed composed of home-grown cereals (50–55% barley, 25–30% wheat, 10% peas), 7% soy-bean meal, and a 2-% commercial concentrate including amino acids, vitamins and minerals, mixed with water. Feed was provided as a”phase-1-feed” including a high protein content (~17–19% crude protein) until the pigs had reached a live weight of approximately 60–70 kg, and thereafter a “phase-2-feed” with a slightly lower protein content (16–18% crude protein) and slightly more water. The total dry matter was similar throughout. At arrival, the total energy provided per day was 15–16 MJ, being successively increased to 34 MJ, thereafter being maintained at 34 MJ to avoid excess fat deposition. The farmer considered the period following this detaining of energy to be the critical time period for tail biting. The slaughter commenced approximately 10 weeks after arrival and when fewer than eight pigs remained in the pen, the energy content was increased to 35 MJ (Hampshire crossbreds) or to 37 MJ (Duroc crossbreds). The total MJ/day and the amounts of protein in the feed did not change between Period 1 and 2. However, eight months after the change in feeding regime, the source of protein was changed from a mixture of protein sources (rapeseed meal, soybean meal and distillers’ dried grain) to pure soybean protein.

### Productivity data

The farm used the system “PigWin Slaughter” (Swedish Animal Health Service, Uppsala, Sweden) for recording of production data. The production data included all pigs that arrived and were sent to slaughter within the respective periods 1 and 2 (Table 1). All treatments were manually recorded in farm-specific records. The recordings registered as the code 257/258 referring to “tail lesion” at slaughter were made available from the advisory company “Swedish Animal Health Service” and included in “PigWin Slaughter”.

**Table 1:** The total number of pigs included in investigations for the respective parameter measured and in each of the two periods; before (Period 1; 2008–04-01 to 2009–06-30) and after (Period 2; 2010–01-01 to 2011–03-31) a change of the feeding regime in a finisher herd

Parameters measured	Period 1	Period 2	Total
Remarks at slaughter	6390	9478	15868
Treatment records	6378	8724	15102
Average daily weight gain	6283	9478	15761
Feed conversion ratio	6283	9478	15761

### Health records

The farm practised”conditional medical treatment”, a practise that is regulated by law and implies that animal caretakers, following a regulated course-work and under the supervision of the herd veterinarian, are allowed to induce specified treatments upon specified clinical signs (The Swedish Board of Agriculture, No 2023:21, D35/L41, www.jordbruksverket.se).

The pigs subjected to antibiotic treatment for tail biting and thus included in the records were those where more than half of the tail was missing, or the tail was red and swollen, or the tail was bitten and the pig appeared dull and/or had fever. Treatment included the use of penicillin G procaine i.m. for 3–5 days. In all cases of tail lesions, the tip of the tail was covered with tar.

### Statistics

The number of pigs getting a remark for the code 257–258 at slaughter was calculated as the percentage of pigs out of the total number of pigs slaughtered during the same period and the difference between Period 1 and 2 was calculated using a Chi-square test.

The number of pigs treated for tail lesions were calculated as the percentage of pigs treated out of the total number of pigs present in the stables during the same period and the difference between Period 1 and 2 was calculated using a Chi-square test.

The difference in average daily weight gain between Periods 1 and 2 were calculated using an analysis of variance (ANOVA) including stable and batch effects.

The difference in average feed conversion ratio between Periods 1 and 2 were calculated using an analysis of variance (ANOVA) including stable and batch effects.

## Results

In total 15,868 slaughtered pigs were included in the study, 6390 pigs in Period 1 and 9478 in Period 2 (Table 1). Some treatment records and some registrations of weight and feed consumptions were missing, hence the numbers of animals included in each variable may vary.

**Fig. 1:**
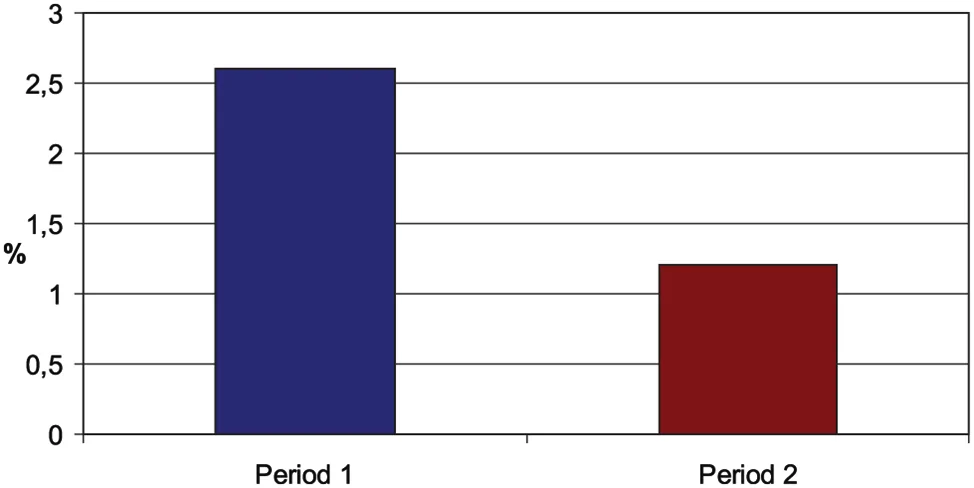
The proportion of pigs registered for tail lesions at slaughter, defined as ”if half or less of the tail is left” or “if an inflammatory reaction is at hand” in ”Period 1” (before the change in feeding regime) and in ”Period 2” (after the change in feeding regime); *p* < 0,001

The average number of remarks for tail lesions at slaughter was reduced from 147 pigs (2.3% of the total number slaughtered) during Period 1, to 113 pigs (1.2%) during Period 2, a reduction by 54% (*p* < 0.001; Fig. 1).

The number of pigs subject to antibiotic treatment for “tail biting” included pigs that had arrived, were treated, and slaughtered within the same period, and for whom treatment data were recovered, among a total of 15,102 animals. The number of pigs treated was reduced from 0.9% (59 of 6378 pigs) in Period 1, to 0.6% (52 of 8724 pigs) in Period 2, a reduction by 35% (*p* < 0.01).

The number of pigs for which the daily weight gain and feed conversion ratio were measured included pigs that had arrived, were treated, and slaughtered within the same period, and for whom data on growth performance were registered in “PigWin slaughter” (Table 1). The average DWG when the pigs were fed three times daily was 790 g/day and when they were fed four times a day, the DWG was 830 g/day, an increase by 46/day (*p* < 0.0007). The feed conversion ratio as measured by MJ/kg growth was 38.5 MJ/kg when the pigs were fed three times daily, and 37.2 MJ/kg growth when they were fed four times daily, a reduction of 1.2 MJ/kg growth/day (*p* < 0.024).

## Discussion

Changes in management may have profound effects for the animals. The present investigation was undertaken because a decrease in the frequency of tail biting was consistently noted at the routine herd inspections. An interview with the foreman gave at hand, that the number of feeding occasions per day had been increased, whereas no other, obvious changes in the management had been undertaken. Scrutinizing the herd records confirmed this information.

Several studies mention feeding strategies and feed composition as a possible underlying cause of tail biting [4, 6, 20, 21, 23] but few studies have addressed the number of feeding occasions per day. In a survey by Valros et al. [9], farmers rated “feeding-related issues such as enough feeding times” to be among the most important preventative measures. Contradicting our findings, Hessel et al. [26] however displayed more aggressive behaviour at 18 w. of age and greater skin lesion scores on the head and body at 28 w. of age, when comparing feeding three times a day to feeding nine times a day in 28 “focal pigs” being observed during the morning feeding in weeks 4, 10, and 14. However, the occurrence of tail biting was not reported in that study. Further, a questionnaire survey by Kallio et al. found that feeding the pigs > 2 meals per day but not to appetite, increased the risk of tail biting [27].

A few studies also mention “gut fill” and “satiety” as important factors [4, 18, 23, 25, 27]. Restricted feeding during the second half of the fattening period, aiming to achieve lean carcasses, has been suggested as a risk factor [23], although others have not been able to confirm this [6]. Heritable predisposal for tail biting related to breeding goals promoting lean carcasses have been suggested [13, 17]. Further, highly digestible, low-fibre, or finely grinded pelleted feed [4, 6, 25] have also been identified as risk factors, possibly resulting in the animals not being satiated.

Most authors agree that provision of straw is one of the most important preventive factors [11, 12, 18, 24, 26, 28]. Opinions on the underlying reasons may vary but mostly refer to the ability to perform behavioural needs [4]. However, some authors also mention that the consumption of straw to meet the need for gut fill and satiation may play a role [4, 25, 27]. In the present study, the pigs were provided with 1-kg straw daily later during rearing. However, this routine did not change from Period 1 to 2 and could not serve as an explanation to the decrease in remarks at slaughter and in the treatments for tail biting.

Feeding space may also influence the frequency of tail biting [6, 25] since it will affect the competition for feed [25]. Even though an automatic feeder will provide feed *ad lib*, the number of feeding places is important since feeding is a socially facilitated behaviour, and subordinate pigs may not be able to maintain their *ad lib* intake [20, 23]. Similarly, a high stocking density may increase the risk for tail biting [12, 18]. The stocking density increases with age, and the risk thus relates to both the competition for food and the restricted feeding applied in some herds [23]. In the present study, the pigs had acquaintance to a total area of 0.77–0.85 m^2^ per pig, an area that has not previously been shown to have any significant effect on its own [12]. The space available at the feeding through was slightly less than the 33 cm reported by others [26]. However, these parameters were neither changed between Period 1 and Period 2.

Taken together, feed seems to be an important factor in the development of tail biting. Purely speculatively, the provision of straw that act as a bulk material and to some extent also is fermented and digested in the large intestine, accounting for >80% of the digesta transit time, may induce a sense of satiety. Pigs are hind-gut fermenters and, as also suggested by Schrøder-Petersen & Simonsen [4], pigs may remain hungry after a meal when fed a low-fibre diet. This might offer a plausible explanation for the results in the present study: Increasing the number of feeding sessions decreases the interval between each feeding, thereby increasing the time that the gut is filled, and the time that the pig feels sated. However, as discussed by Kallio et al., if the pigs are fed many separate, small meals they might still be hungry afterwards [27].

Sweden is one of the few countries where tail docking is never performed. The reported prevalence of tail lesions is 1.5 - 1.9%, as being monitored at the abattoir. In the present study, 2.3% before the change in feeding regimen must be regarded as high, whereas the 1.2% in Period 2 is below average. Official meat inspection records typically show much lower prevalence than the results from more detailed investigations performed in specific studies [28]. Thus, figures based on official records probably constitutes a bias, since the criterion for getting a remark is”if half or less of the tail is left” or if an inflammatory reaction is at hand [15]. Thus, small lesions will be overlooked, whereas unrelated lesions such as tail necrosis, will be included. Further, discrepancies between slaughterhouses are evident. However, since the same slaughterhouses were employed during Period 1 and 2, a comparison within this herd will still be valid. Nevertheless, figures based on this criterion cannot be compared to figures from countries practising tail docking [2, 3].

The treatment regimens performed on this farm was based on recommendations by the herd veterinarian and did not include the use of non-steroidal antiphlogistic drugs. However, the appropriate use of such drugs may be advocated.

The economic losses caused by tail biting include condemnation of carcasses at slaughter, increased losses due to euthanization and mortality at the farm, a decrease in daily weight gain, an increase in feed conversion ratio, an increase in the number of feeding days, increased costs for treatment, and a reduction in payment at slaughter caused by a reduction of secondary infections such as abscesses [5]. Thus, simple measures such as increasing the number of feeding occasions per day may be well worth trying in order to reduce the frequency of lesions, also from an economic point of view.

The main limitations of this study include the fact that the pigs were crossbred, including boars of different genetics, and that piglets originated from farms with different health status, both factors that reportedly may affect the frequency of tail-biting [17, 18]. Further, the recordings at slaughter were performed by different veterinary inspectors and this may also have affected the results, although the assessments supposedly should be performed according to the same regulations. However, these factors varied randomly throughout the study and were thus present both before and after the change in feeding regime. Although the total amount of protein was the same, it is further not known if the change in protein source may have affected the results. There may also be an effect of the season. All months of the year were included both before and after the change, however, since each period included 15 months, some months were included twice in the recordings. The average outdoor temperatures in these overlapping months were +7.6, +11.2 respective +13.0 °C in “Period 1”, and −3.7, −5.4 and −0.5 °C, respectively, in “Period 2”, and although none of these temperatures are to be regarded as extreme, it may have affected the indoor climate to some extent. Further, more variables could have been included in the statistical calculations, but some data were not possible to retrieve.

## Conclusion

An increase in the number of feeding bouts from three to four times daily seems to reduce the frequency of tail biting, as monitored by the number of remarks at slaughter and the number of treatments for tail biting. Further, the increase resulted in a higher average daily weight gain and in a decrease in the feed conversion ratio.

## Data Availability

All data are available at the Swedish University of Agricultural Sciences upon request.
